# AVP-GPT2: A Transformer-Powered Platform for De Novo Generation, Screening, and Explanation of Antiviral Peptides

**DOI:** 10.3390/v17010014

**Published:** 2024-12-25

**Authors:** Huajian Zhao, Gengshen Song

**Affiliations:** Beijing Youcare Kechuang Pharmaceutical Technology Co., Ltd., Beijing 100176, China; zhaohuajian@youcareyk.com

**Keywords:** antiviral peptide, star-transformer, vision-transformer, generation model, antiviral activity screening model, toxicity screening model, SHAP

## Abstract

Human respiratory syncytial virus (RSV) remains a significant global health threat, particularly for vulnerable populations. Despite extensive research, effective antiviral therapies are still limited. To address this urgent need, we present AVP-GPT2, a deep-learning model that significantly outperforms its predecessor, AVP-GPT, in designing and screening antiviral peptides. Trained on a significantly expanded dataset, AVP-GPT2 employs a transformer-based architecture to generate diverse peptide sequences. A multi-modal screening approach, incorporating Star-Transformer and Vision Transformer, enables accurate prediction of antiviral activity and toxicity, leading to the identification of potent and safe candidates. SHAP analysis further enhances interpretability by explaining the underlying mechanisms of peptide activity. Our in vitro experiments confirmed the antiviral efficacy of peptides generated by AVP-GPT2, with some exhibiting EC50 values as low as 0.01 μM and CC50 values > 30 μM. This represents a substantial improvement over AVP-GPT and traditional methods. AVP-GPT2 has the potential to significantly impact antiviral drug discovery by accelerating the identification of novel therapeutic agents. Future research will explore its application to other viral targets and its integration into existing drug development pipelines.

## 1. Introduction

Lower respiratory infections are a leading cause of death worldwide, especially among infants. Human respiratory syncytial virus (RSV), a major contributor to this global health crisis, causes severe respiratory infections in infants, young children, the elderly, and immunocompromised individuals. Globally, RSV is responsible for approximately 60,000 in-hospital deaths annually in children under five years old. Despite extensive research, effective antiviral therapies for RSV remain limited, with most treatment options focused on supportive care [1,2,3,4,5].

The RSV fusion glycoprotein (F) is a key target for antiviral drug development. Peptides, with their ability to be tailored to target specific viral proteins, present a promising opportunity for developing targeted therapies. Peptides derived from the HR1 and HR2 domains of RSV F can inhibit viral entry by preventing the formation of the six-helix bundle hairpin structure. Previous research has investigated the use of synthetic peptides from the RSV HR2 domain as potential antiviral agents. By scanning synthetic peptides of 35 amino acids in length across the RSV HR2 wild-type sequence, researchers have identified peptides, such as T118 with an EC50 of 0.05 µM, that can block virus-induced cell fusion. These findings suggest that targeting the HR2 domain could be a promising approach for developing antiviral therapies [1,2]. While extensive research has been conducted, effective antiviral therapies for RSV remain elusive. The traditional approach of identifying effective antiviral peptides is often labor-intensive and costly.

Machine learning, particularly that based on transformer models, has emerged as a powerful tool for accelerating drug discovery. Transformers, leveraging the attention mechanism, can effectively analyze sequential data, such as peptide sequences. This capability enables the identification of complex patterns and relationships, which is crucial for designing and screening potent antiviral peptides. Our previous work, AVP-GPT, demonstrated the potential of transformer-based models in this domain, identifying four peptides against RSV with EC50 values as low as 0.02 µM, the strongest reported to date [6].

Deep-learning models inspired by transformers, including Star-Transformer and Vision Transformer (ViT), have gained popularity in various fields and achieved impressive results. Star-Transformer, a simplified version of the Transformer model, addresses its computational complexity by using a sparse topology. It replaces the fully connected structure with a star topology, reducing the model’s complexity from quadratic to linear while maintaining its ability to capture local relationships and long-term dependencies. Compared to the Transformer model, Star-Transformer is better suited for smaller datasets. While Star-Transformer has been successfully applied to MHC I allele binding peptide prediction, the application of these latest models to peptide design remains unexplored [7]. ViT, a pure Transformer applied directly to sequences of image patches, is a highly effective model for image classification, surpassing state-of-the-art convolutional networks while requiring significantly fewer computational resources for training [8]. However, its application to peptide design has not been investigated.

In this study, we introduce AVP-GPT2, a novel deep-learning model specifically designed for antiviral peptide design. AVP-GPT2 builds upon AVP-GPT, incorporating advanced transformer-based architectures. Trained on a larger dataset, AVP-GPT2 captures complex peptide-receptor relationships, enabling the generation of specific antiviral candidates. By incorporating Star-Transformer and Vision Transformer architectures, AVP-GPT2 enhances peptide screening efficiency and accuracy. Additionally, SHAP analysis helps interpret peptide activity, guiding the design process toward more effective antiviral agents. By understanding the factors that contribute to a peptide’s antiviral efficacy, we can further refine the design process and accelerate the development of novel therapeutic agents. These innovative approaches have the potential to significantly impact the discovery of potent antiviral agents against RSV and other viruses.

## 2. Materials and Methods

### 2.1. Datasets

To create a comprehensive dataset for antiviral peptide design and screening, we merged sequences from three sources: our in-house Youcare dataset, the publicly available AVPdb [9], and the DRAVP database [10] (Supplementary Tables S1 and S2). The Youcare dataset consists of manually designed peptides targeting RSV, INFV, and SARS-CoV-2, while AVPdb and DRAVP contain a broader collection of antiviral peptides against various viruses. After removing redundant sequences, our pre-processed dataset for antiviral activity included 1657 peptides targeting HIV, RSV, INFV, HCV, SARS-CoV, SARS-CoV-2, FIV, HSV, and HPIV. Peptides with reported IC50/EC50 values below 1 μM were classified as active (label of “1”), while those with IC50/EC50 values equal to or greater than 1 μM were considered inactive (label of “0”). For toxicity screening, we included 713 non-redundant peptides from the same viruses. Peptides with CC50 values below 30 μM were classified as toxic (label of “1”), while those with CC50 values above 30 μM were considered non-toxic (label of “0”).

### 2.2. Sequence Tokenization

Sequence tokenization was performed by treating each amino acid and peptide modification as an independent token. This resulted in a total of 25 tokens, including 20 amino acid tokens (A, R, N, D, C, Q, E, G, H, I, L, K, M, F, P, S, T, W, Y, and V), 3 peptide modification tokens (<M1>, <M2>, and <M3>), and 2 special tokens (<sep> and <unknown>). AVP-GPT2 receives both receptor and peptide tokens, with a maximum input length of 949. The receptor sequence and peptide sequence were concatenated, with the receptor sequence placed before the peptide sequence and a <sep> token used to separate the two. This tokenization scheme allows AVP-GPT2 to effectively process both receptor and peptide information for antiviral peptide design.

### 2.3. Structure Prediction

To analyze the three-dimensional conformation of peptides and receptors, we predicted their structures using the HelixFold-Single tool [11]. HelixFold-Single utilizes a protein language model to predict protein structures without the need for multiple sequence alignments. The predicted structures were then visualized using PyMOL Open-Source 2.5.0 (https://github.com/schrodinger/pymol-open-source, accessed on 11 June 2024), a popular open-source software for molecular visualization. Finally, the predicted structures were saved in PNG format with a resolution of 224 × 224 pixels for further analysis.

### 2.4. Peptide Descriptors

To characterize peptides, we computed 18 protein features using Pfeature [12]. These features include Amino acid composition (AAC), Dipeptide composition (DPC), Tripeptide composition (TPC), Atomic composition (ATC), Bond composition (BTC), Physico-chemical properties composition (PCP), Residue repeat information (RRI), Physico-chemical properties repeat information (PRI), Distance distribution of residues (DDR), Shannon entropy of protein (SEP), residues (SER), physicochemical properties (SPC), Conjoint triad descriptors (CTC), Composition enhanced transition distribution (CeTD), Pseudo amino acid composition (PAAC), Amphiphilic pseudo amino acid composition (APAAC), Quasi sequence order (QSO), and Sequence order coupling number (SOC). In total, 9189 peptide descriptor features were generated. To identify the most informative features, we applied Lasso feature selection, a regularization technique that helps prevent overfitting by penalizing features with small coefficients [13]. The selected features were subsequently fed into baseline machine learning models, including Random Forest (RF) and Support Vector Machine (SVM), for both antiviral activity and toxicity screening.

### 2.5. Model Architecture

AVP-GPT2 consists of four main components: a generation module, an antiviral activity screening module, a toxicity screening module, and an explanation module. The generation module architecture includes an input embedding layer that processes receptor and peptide tokens, four transformer decoder layers (each containing layer normalization and residual connections to improve training stability and gradient flow, a masked multi-head attention layer with three heads that capture dependencies within the sequence ability and gradient flow, a layer normalization and residual connections, and a feed-forward neural network (MLP) for non-linear transformations), and a linear and softmax layer to generate a probability distribution over the output vocabulary.

The screening modules in AVP-GPT2 consist of a star-transformer and MLP layer to process receptor and peptide sequences, two ViTs to capture the structural features of receptor and peptides, a transformer encoder to fuse the vision information from the ViTs, and multiple MLP layers for classification. The structural features were split into different patches, flattened, linearly projected, combined with positional embedding, and fed into a transformer encoder. This process allows the model to capture the structural features and effectively incorporate them into the prediction process. The activity screening module evaluates the potential antiviral activity of generated peptides, while the toxicity screening module assesses their toxicity profiles. The star transformer used in the screening modules has one layer and eight heads, while the MLP output dimension is 256, connected to the star transformer. A patch size of 8 × 8 pixels for receptor structure and a patch size of 16 × 16 pixels for peptide structure were used. The two ViTs used for receptor and peptide structure have two layers and two heads, with an MLP output dimension of 256 connected to the transformer encoder.

The explanation module consists of a Star Transformer model, where only the peptide sequence is fed as input. To understand the factors contributing to antiviral and toxicity predictions, we employed the SHAP (SHapley Additive exPlanations) method [14]. SHAP provides a feature attribution approach that quantifies the contribution of each feature to the model’s prediction. By applying SHAP to the peptide sequences processed by the Star Transformer model, we were able to identify the specific amino acids that were most influential in determining antiviral and toxicity outcomes. This analysis allowed us to gain insights into the underlying mechanisms of action for the generated peptides.

### 2.6. Training Setup

The data was split into training (60%), validation (20%), and testing (20%) sets for AVP-GPT2, and all models were trained using the AdamW optimizer [15] with a batch size of 32 for 500 epochs on an NVIDIA^®^ V100 Tensor Core GPU.

### 2.7. Evaluation Methods

The perplexity was used to compare AVP-GPT2’s generation performance to a LSTM model, a common approach for peptide generation. Lower perplexity indicates better performance [16,17]. The AUC (Area Under the Curve) was used to evaluate the classification performance of AVP-GPT2 compared to a RF and SVM model, widely used methods for antiviral activity and toxicity prediction [18]. Higher AUC signifies better classification ability. Precision, recall, and F1-score were used to assess the classification performance in terms of the model’s ability to correctly identify positive cases (precision and recall) and to balance these two metrics (F1-score).

### 2.8. Software Implementation of AVP-GPT2

AVP-GPT2 was implemented using Python 3.11.7 on a CentOS Linux 7.2 server with PyTorch 2.1.2 [19] and scikit-learn 1.4.0 [20] for deep-learning functionalities.

### 2.9. In Vitro Studies of RSV

As described by our previous report [6], the identified AVP candidates were synthesized using solid-phase peptide synthesis (SPPS) [2] by Chinese Peptide Company (CPC), analyzed by UPLC/MS to verify their purity and molecular weight, purified by HPLC preparative to achieve high purity levels, and sequenced to confirm the correct amino acid sequence. The antiviral activity of the synthesized peptides against RSV was evaluated by WuXi AppTec using a plaque reduction assay with the RSV strain A Long, with HEp-2 cells infected with RSV and treated with serially diluted peptides, followed by quantification of viral replication using RSV-specific antibodies and a secondary antibody. Cytotoxicity experiments were conducted in parallel with antiviral experiments using HEp-2 cells, with cell viability detected using the CCK8 assay [6].

## 3. Results

### 3.1. Workflow of the AVP-GPT2

**Figure 1:**
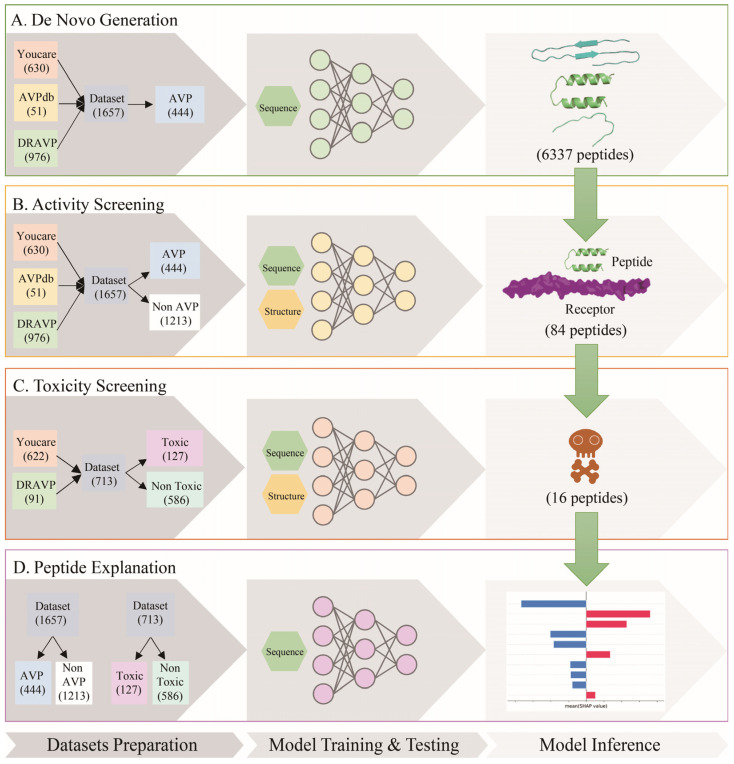
AVP-GPT2 workflow for AVP generation, screening, and explanation. The AVP-GPT2 workflow comprises four key stages: de novo generation, activity screening, toxicity screening, and peptide explanation. (**A**). De Novo Generation: A GPT model generates novel peptide sequences based on reference viral sequences. (**B**). Activity Screening: A multimodal model evaluates the antiviral activity of generated peptides using sequence and structure information. (**C**). Toxicity Screening: Another multimodal model assesses the toxicity of identified AVPs. (**D**). Peptide Explanation: SHAP analysis identifies key features associated with antiviral activity and toxicity. A total of 6337 peptides were generated, 84 were identified as AVPs, and 16 of these were predicted as non-toxic. The 16 non-toxic peptides were further refined based on SHAP analysis to improve antiviral activity and decrease toxicity. Each stage involves three processes: dataset preparation, model training and testing, and model inference. Datasets collected from our group (Youcare), AVPdb and DRAVP were classified as AVP and Non AVP with the cut-off IC50/EC50 1 μM. Of the 1657 data, 444 were labeled as AVP, and 1213 were labeled as Non AVP. Only the AVPs were fed into the de novo generation model to learn the rules of AVPs, while both peptides with or without antiviral activity are used in the activity screening multimodal model to learn how to identify AVP. A total of 713 data from our group and DRAVP with toxicity information were classified as toxic and non-toxic with the cut-off CC50 30 μM. 127 peptides were labeled as toxic, and 586 peptides were labeled as non-toxic. Both peptides with or without toxicity are used in the toxicity screening multimodal model to learn how to identify toxicity.

The AVP-GPT2 workflow comprises four key stages: de novo generation, activity screening, toxicity screening, and peptide explanation (Figure 1). In the de novo generation stage, a GPT model was trained to generate novel peptide sequences using the reference sequences of different viruses as input. The activity screening stage employed a multimodal model utilizing sequence and structure information to identify antiviral peptides (AVPs) from the generated pool. Another multimodal model was used in the toxicity screening stage to assess the toxicity of the identified AVPs. Finally, SHAP analysis was performed to identify the key features associated with antiviral activity and toxicity in the peptide explanation stage.

In the model inference process, a total of 6337 peptides targeting RSV were generated by the GPT model using the RSV HR1 reference sequence (NCBI accession FJ614815) as input. A total of 84 peptides were identified as AVPs, and 16 of these were identified as non-toxic and further refined based on SHAP analysis to improve antiviral activity and decrease toxicity. The refined peptides were confirmed to be antiviral and without toxicity by AVP-GPT2 screening parts.

### 3.2. Datasets Preparation

**Figure 2:**
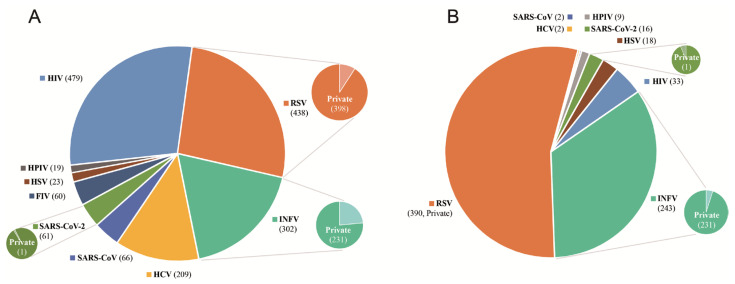
The distribution of antiviral peptides and toxicity information. (**A**). Distribution of antiviral peptides Across Different Viruses. This pie chart illustrates the distribution of antiviral peptides (AVPs) and non-AVPs within the dataset, categorized by the targeted virus. The number of peptides for each virus is indicated in parentheses. The dataset is enriched with peptides targeting HIV, RSV, and INFV, which are known to be significant public health concerns. (**B**). Distribution of Toxic and Non-Toxic Peptides Across Different Viruses. This pie chart depicts the distribution of toxic and non-toxic peptides within the dataset, categorized by the targeted virus. The dataset is enriched with peptides targeting RSV and INFV, primarily from our private Youcare dataset.

To construct a comprehensive dataset for antiviral peptide design and screening, we combined sequences from our in-house Youcare dataset with publicly available data from AVPdb and DRAVP. The dataset consisted of 1657 non-redundant sequences targeting various viruses, including HIV, RSV, INFV, HCV, SARS-CoV, SARS-CoV-2, FIV, HSV, and HPIV, expanding our previous dataset (604 sequences) from AVP-GPT report [6]. The distribution of peptides across different viruses is shown in Figure 2A. As depicted, the dataset is enriched with peptides targeting HIV, RSV, and INFV, which are known to be significant public health concerns. For toxicity screening, we included 713 non-redundant sequences from the same viruses. The distribution of toxic and non-toxic peptides is also shown in Figure 2B. The dataset is enriched with peptides targeting RSV and INFV, primarily from our group (labeled as Private). This diverse dataset provided a robust foundation for training and evaluating our AVP-GPT2 model. It ensured that the model was exposed to a wide range of peptide sequences targeting different viruses, improving its ability to generalize and identify antiviral peptides against diverse targets.

### 3.3. De Novo Generation

**Figure 3:**
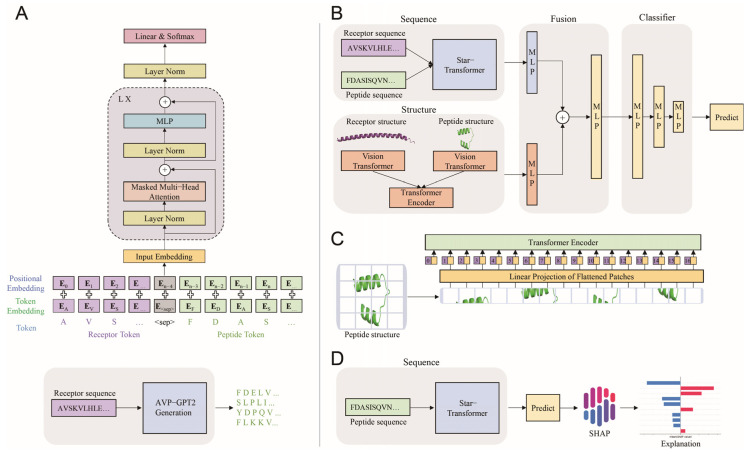
AVP-GPT2 Model Architecture with Key Features. (**A**). De novo generation module architecture. The generation module in AVP-GPT2 consists of an input embedding layer, layer normalization, residual connections, a masked multi-head attention layer, a feed-forward neural network (MLP), and a linear and softmax layer. Receptor and peptide tokens are processed sequentially, with a special <sep> token separating them. Positional embeddings are added to the input tokens before being fed into the transformer encoder. The output of the transformer encoder is connected to the MLP layer, followed by the linear and softmax layer to generate the final peptide sequence. By providing a specific receptor sequence as input, the generation module can design novel peptide sequences tailored to the receptor’s characteristics. (**B**). Activity and toxicity screening module architecture. The screening modules in AVP-GPT2 receive sequence and structural features as input. A star-transformer processes the receptor and peptide sequences, while two ViTs capture the structural features. A transformer encoder fuses the vision information extracted from the ViTs. The fused features are then passed through multiple MLP layers for classification. This architecture effectively integrates sequence and structural information to accurately predict both antiviral activity and toxicity. (**C**). ViT architecture for screening modules. The ViT architectures in the screening modules process peptide or receptor structure images by dividing them into a patch size of 16 × 16 pixels or 8 × 8 pixels, flattening the patches, linearly projecting them, adding positional embeddings, and finally feeding them into a transformer encoder. This approach allows the model to capture the structural features of peptides and receptors. (**D**). Explanation Module Architecture. The explanation module in AVP-GPT2 utilizes the Star-transformer model to predict antiviral activity or toxicity for peptide sequences. SHAP analysis is then applied to identify specific amino acid sequences associated with these activities or toxicities. By analyzing the SHAP values, the model can determine which amino acids contribute most significantly to the predicted antiviral or toxic effects. Note: The full sequences of the receptor and peptide are not shown in their entirety due to space constraints. The ellipsis (...) indicates the omitted portion of the sequence.

The maximum input length for the AVP-GPT2 generation module was 949, considering the maximum length of receptors (852) and peptides (96) with the addition of the <sep> token (Figure 3A). To decrease the number of parameters for training, the input embedding layer and the linear layer connected to the transformer decoder share weights. Additionally, flash attention is used to replace attention, speeding up the training process.

**Figure 4:**
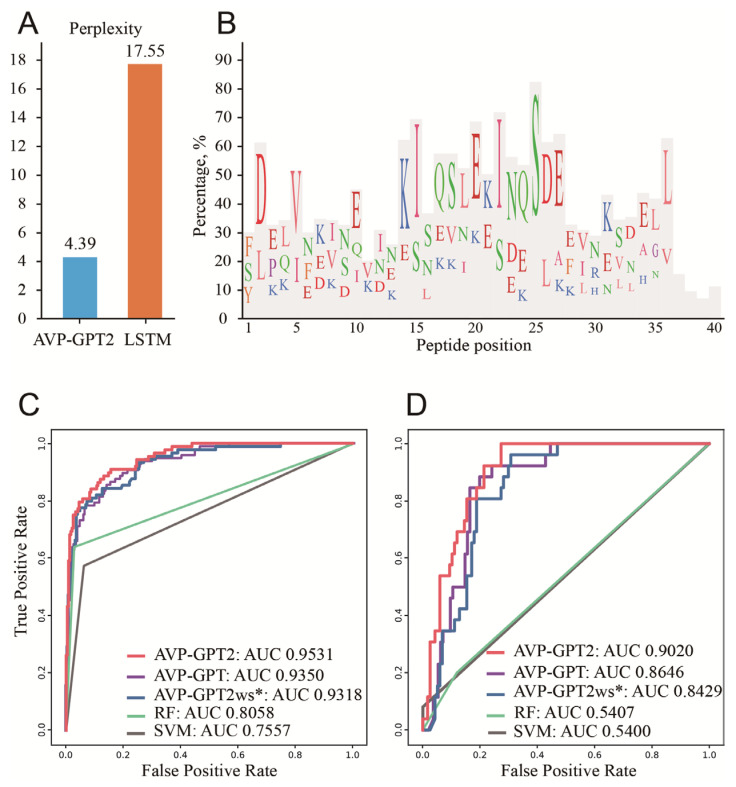
Evaluation of AVP-GPT2’s antiviral peptide generation and screening. (**A**). Perplexity comparison of AVP-GPT2 and LSTM. Perplexity is a measure of how well a model can predict the next token in a sequence. A lower perplexity indicates better performance. (**B**). Amino acid composition of generated peptides. This bar chart illustrates the distribution of amino acids at different positions within the 6337 peptides generated by AVP-GPT2. The *x*-axis represents the peptide position (1–40), and the *y*-axis represents the percentage of occurrence for each amino acid at that position. The chart highlights the most common amino acid sequences (with a frequency greater than 10%) observed at each peptide position. (**C**). Antiviral Activity Screening: This ROC curve compares the performance of AVP-GPT2, AVP-GPT2 without structural information (AVP-GPT2 ws), AVP-GPT, Random Forest (RF), and Support Vector Machine (SVM) models in antiviral activity screening. The ROC curve plots the true positive rate against the false positive rate for different classification thresholds. An ideal ROC curve would hug the top-left corner, indicating perfect classification. The AUC quantifies the ability of the model to distinguish between AVPs and non-AVPs. A higher AUC indicates that the model is better at distinguishing between peptides that exhibit antiviral activity and those that do not. AVP-GPT2 exhibits a higher AUC score compared to AVP-GPT, AVP-GPT2 ws, RF, and SVM, demonstrating their superior ability to distinguish between antiviral and non-antiviral peptides. (**D**). Toxicity Screening: This ROC curve compares the performance of AVP-GPT2, AVP-GPT2 ws, AVP-GPT, RF, and SVM models in toxicity screening. Similar to panel C, the ROC curve plots the true positive rate against the false positive rate for different classification thresholds. AVP-GPT2 exhibits a higher AUC score compared to AVP-GPT, AVP-GPT2 ws, RF, and SVM, demonstrating its superior ability to distinguish between toxic and non-toxic peptides.

The AVP-GPT2 generation part achieved a remarkably lower perplexity (4.39) compared to a well-known generative model for peptides, LSTM (perplexity: 17.55) (Figure 4A). This result suggests that AVP-GPT2 can generate peptide sequences with greater confidence and potentially higher quality. A total of 6337 unique RSV peptides were successfully generated, with a maximum length of 40 amino acids. The most common amino acids observed at single peptide positions were F, S, Y, D, L, E, P, K, Q, V, I, N, A, R, H, and G (with a count percentage greater than 10%) (Figure 4B). While this study focused on RSV, AVP-GPT2 could be applied to generate peptides targeting other viruses by simply providing different reference sequences, demonstrating its versatility and potential for broad applications in antiviral peptide design.

### 3.4. Activity Screening

The screening modules in AVP-GPT2 handle sequence and structure information (Figure 3B,C). AVP-GPT2 demonstrated exceptional performance in antiviral peptide screening, achieving an AUC of 0.9531, precision of 0.8876, recall of 0.8818, and F1-score of 0.8598 (Figure 4B, Table 1). These metrics significantly outperformed our previous AVP-GPT and state-of-the-art models, RF and SVM, underscoring AVP-GPT2’s ability to effectively distinguish AVPs from non-AVPs. The model’s effectiveness in capturing the intricate relationships between peptide sequences, structures, and antiviral activity was evident in its high AUC, precision, recall, and F1-score values. Notably, the removal of structural information from AVP-GPT2 led to a significant decrease in performance, emphasizing the crucial role of incorporating structural features in achieving superior prediction accuracy. This suggests that AVP-GPT2 can effectively leverage both sequence-based and structure-based information to enhance prediction performance. From the 6337 unique RSV peptides generated, the AVP-GPT2 classification component identified 84 peptides with a high probability (>0.9) of being AVPs. These top candidates were selected for further toxicity prediction.

### 3.5. Toxicity Screening

The AVP-GPT2 activity and toxicity screening modules share the same architecture, utilizing sequence and structure information as input. AVP-GPT2 demonstrated superior toxicity prediction capabilities, achieving an AUC of 0.9020, precision of 0.7545, recall of 0.7444, and F1-score of 0.7492 (Figure 4C, Table 2). These results significantly outperformed our previous AVP-GPT and state-of-the-art models, RF and SVM. The incorporation of structural information within AVP-GPT2 was crucial for its performance, as evidenced by the decline in metrics when this information was removed. From the 84 unique RSV potential AVPs, 16 were identified as non-toxic with high probability (>0.9) and selected for further adjustment based on the peptide explanation results.

### 3.6. Peptide Explanation

We utilized SHAP to identify specific amino acid sequences associated with antiviral and toxic activities by analyzing the peptide sequences processed by the Star Transformer model. By selecting the specific classification (1: antiviral for activity screening data, 1: toxicity for toxicity screening data), we were able to gain a deeper understanding of the relationship between peptide sequence and antiviral and toxic activities. To comprehensively analyze this relationship, the entire dataset was fed into the Star Transformer model.

**Figure 5:**
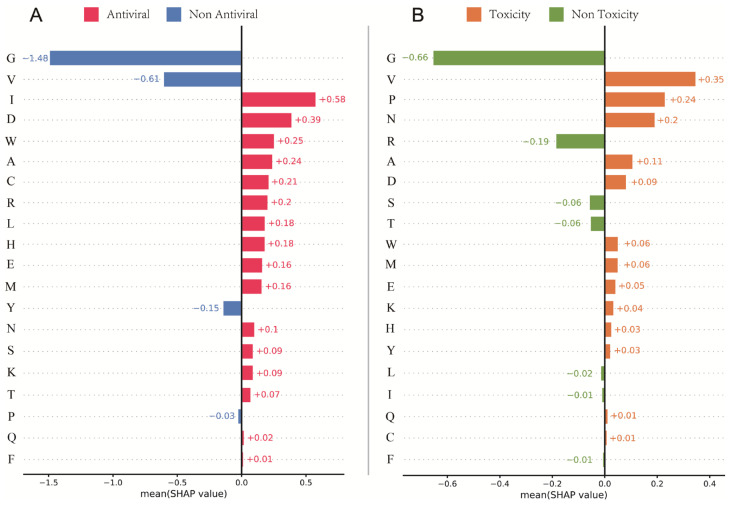
SHAP Analysis for antiviral and toxicity prediction. (**A**). SHAP Analysis for antiviral activity. This bar chart illustrates the SHAP values for individual amino acids, indicating their contribution to the prediction of antiviral activity. Positive SHAP values suggest that the amino acid is associated with antiviral activity, while negative SHAP values suggest an association with non-antiviral activity. The absolute value of the SHAP value represents the magnitude of the influence of the amino acid on the prediction. (**B**). SHAP analysis for toxicity. This bar chart illustrates the SHAP values for individual amino acids, indicating their contribution to the prediction of toxicity. Positive SHAP values suggest that the amino acid is associated with toxicity, while negative SHAP values suggest an association with non-toxicity.

The SHAP analysis revealed that specific amino acid sequences were associated with antiviral and toxic activities. Peptides containing amino acids I, D, W, A, C, R, L, H, E, M, N, S, K, T, Q, and F were more likely to exhibit antiviral activity, while peptides containing amino acids G, V, Y, and P were more likely to be non-antiviral (Figure 5A). Conversely, peptides containing amino acids V, P, N, A, D, W, M, E, K, H, Y, Q, and C were more likely to exhibit toxicity, while peptides containing amino acids G, R, S, T, L, I, and F were more likely to be non-toxic (Figure 5B). From the 16 unique RSV potential AVPs, sequences associated with non-antiviral and toxic activities were deleted or replaced with amino acid sequences associated with antiviral and non-toxic activities. The adjusted peptides were then further predicted by AVP-GPT2 to validate their antiviral activity and toxicity.

### 3.7. In Vitro Study

The in vitro evaluation of the identified AVP candidates revealed that 14 out of 16 exhibited antiviral activity with EC50 values below 1 μM (Table 3). Notably, ten of these peptides demonstrated exceptional potency with EC50 values around 0.01 μM. This highlights the model’s ability to generate highly effective antiviral candidates. Additionally, the low cytotoxicity of the identified AVPs is promising, with 3 out of 10 candidates exhibiting CC50 (cytotoxic concentration) values exceeding 30 μM, indicating a favorable safety profile. These findings collectively suggest that the three AVPs (AVP-GPT2-6, AVP-GPT2-12, and AVP-GPT2-13) designed by AVP-GPT2 represent promising candidates for further development as anti-RSV therapeutics. AVP-GPT2’s ability to generate antiviral peptides targeting RSV suggests its potential for designing peptides against other viruses, such as INFVA and HPIV.

## 4. Discussion

This study introduces AVP-GPT2, a novel deep-learning model designed to revolutionize the field of antiviral peptide design. By leveraging transformer-based architectures and multimodal integration, AVP-GPT2 significantly outperforms traditional methods in both peptide generation and screening. The model’s ability to accurately predict antiviral activity and toxicity, coupled with its exceptional performance in generating potent peptides, demonstrates its potential to accelerate the development of novel antiviral therapeutics.

Previous peptide generation models like LSTM [21] can suffer from the vanishing gradient problem, limiting their ability to capture long-term dependencies. LSTMs can also be computationally expensive [22,23,24]. In contrast, AVP-GPT and AVP-GPT2’s attention mechanisms mitigate the vanishing gradient issue and enable efficient parallel processing. Both models effectively capture long-term dependencies more effectively than LSTMs. Different from AVP-GPT [6], we significantly expanded our dataset from 604 to 1657 sequences, targeting various viruses. AVP-GPT2 employs specific receptor sequences as input, replacing the “<start>” token. To enhance efficiency, the input embedding layer and linear layer share weights, and flash-attention is used to accelerate training.

Multimodal deep-learning methods, which integrate information from diverse sources, have emerged as a powerful approach in various fields due to their ability to enhance prediction accuracy [25]. By incorporating both sequence and structural data, AVP-GPT2 can leverage a more comprehensive understanding of the underlying biological processes involved in antiviral peptide design. This multimodal approach allows AVP-GPT2 to capture complex relationships and dependencies that may not be apparent when considering only a single modality.

To ensure equal communication between receptor and peptide sequences, even though receptor sequences are generally longer, each token is treated as one length. Given the greater complexity of receptor structures compared to peptide structures, two different patch sizes were used in the ViT models handling these structures. Patches were treated as independent tokens, with a patch size of 8 × 8 pixels for receptor structure and a patch size of 16 × 16 pixels for peptide structure. This approach enables effective communication and analysis of both receptor and peptide structural features. Using self-attention, transformer encoders are adept at fusing and communicating information from different patches. This allows the model to capture the intricate relationships between various structural features and their impact on antiviral activity and toxicity.

Simple models, while often easier to interpret due to their reliance on interpretable features, may compromise accuracy. Deep-learning models, especially transformer-based models, typically exhibit superior accuracy but can be challenging to interpret due to their complex structure and black-box nature [26]. By leveraging SHAP analysis, we were able to bridge this gap, achieving a balance between accuracy and interpretability for our transformer-based model. SHAP allowed us to identify specific amino acids that are closely associated with antiviral activity and toxicity, providing valuable insights into the underlying mechanisms of action. This information enabled us to further refine the peptides designed by AVP-GPT2, potentially enhancing their efficacy and safety profiles.

The peptide T118, derived from the RSV HR2 region, has demonstrated potent anti-RSV activity with an EC50 value of 0.05 μM [27]. Our previous AVP-GPT identified four peptides with an EC50 of 0.02 μM and CC50 > 1 μM. AVP-GPT2 identified three peptides with EC50 values around 0.01 μM and CC50 > 30 μM, demonstrating a 2-fold increase in potency. This highlights AVP-GPT2’s ability to generate highly effective and safe antiviral peptides. AVP-GPT2’s revolutionary approach accelerates the discovery of novel antiviral peptides, addressing the urgent need for new therapies. Its versatility allows for application to other viral targets, expanding its potential impact. AVP-GPT2 represents a significant advancement in antiviral peptide design, with the potential to significantly impact the development of novel antiviral therapeutics. Future research should explore AVP-GPT2’s capabilities and potential applications for addressing other viruses.

## 5. Conclusions

This study introduces AVP-GPT2, a novel deep-learning model that significantly outperforms its predecessor, AVP-GPT, in designing and screening antiviral peptides. Trained on a considerably expanded dataset and incorporating advanced transformer-based architectures, AVP-GPT2 efficiently generates diverse and unique peptide sequences with high confidence, as shown by the lower perplexity compared to LSTM. By integrating sequence and structural information, AVP-GPT2 achieves superior accuracy in identifying antiviral peptides, surpassing AVP-GPT and state-of-the-art models (RF and SVM). The model effectively predicts peptide toxicity with high precision, ensuring the safety of candidate antiviral agents. Furthermore, AVP-GPT2 employs SHAP analysis to explain the key features associated with antiviral activity and toxicity. This allows for targeted refinement of peptide design, leading to potentially more potent and safer antiviral candidates. In vitro experiments confirmed the antiviral efficacy of peptides generated by AVP-GPT2, with some exhibiting exceptional potency (EC50 values as low as 0.01 μM) and excellent safety profiles (CC50 values > 30 μM). This represents a significant advancement over traditional methods and AVP-GPT. AVP-GPT2 has the potential to significantly impact antiviral drug discovery by accelerating the identification of novel therapeutic agents. Future research will explore its application to other viral targets and its integration into existing drug development pipelines.

## Figures and Tables

**Table 1:** Performance comparison of AVP-GPT2 and other models for antiviral prediction. Recall: In the context of our study, recall refers to the model’s ability to correctly identify all true positive instances (i.e., correctly predicted antiviral or toxic peptides). A higher recall value indicates that the model is better at identifying all relevant instances. F1-Score: The F1-score is a harmonic mean of precision and recall, providing a balanced measure of model performance. A higher F1 score indicates that the model has a good balance of precision and recall. Note: AVP-GPT2 ws* = AVP-GPT2 without structure.

Model	Precision	Recall	F1
SVM	0.8149	0.7557	0.7770
RF	0.8856	0.8058	0.8344
AVP-GPT2	0.8876	0.8818	0.8598
AVP-GPT	0.8678	0.8413	0.8529
AVP-GPT2 ws*	0.8605	0.8527	0.8565

**Table 2:** Performance comparison of AVP-GPT2 and other models for toxicity prediction. Note: AVP-GPT2 ws* = AVP-GPT2 without structure.

Model	Precision	Recall	F1
SVM	0.7184	0.5400	0.5297
RF	0.5509	0.5407	0.5434
AVP-GPT2	0.7545	0.7444	0.7492
AVP-GPT	0.5094	0.5021	0.4754
AVP-GPT2 ws*	0.5546	0.5171	0.5033

**Table 3:** In vitro, study results for 16 AVP-GPT2 peptides. EC50: Lower EC50 values indicate greater antiviral potency. CC50: Higher CC50 values indicate lower cytotoxicity.

ID	EC50 (μM)	CC50 (μM)
AVP-GPT2-1	0.011	3.66
AVP-GPT2-2	0.015	6.17
AVP-GPT2-3	2.650	28.34
AVP-GPT2-4	0.029	19.67
AVP-GPT2-5	0.028	30.17
AVP-GPT2-6	0.013	30.68
AVP-GPT2-7	0.010	9.61
AVP-GPT2-8	8.72	35.49
AVP-GPT2-9	0.010	8.90
AVP-GPT2-10	0.023	1.04
AVP-GPT2-11	0.010	17.11
AVP-GPT2-12	0.015	32.57
AVP-GPT2-13	0.013	>100
AVP-GPT2-14	0.010	3.19
AVP-GPT2-15	0.037	28.13
AVP-GPT2-16	0.150	19.29

## Data Availability

To facilitate access to the private dataset, interested researchers can contact us at songgengshen@youcareyk.com or zhaohuajian@youcareyk.com. Upon request, we will provide the dataset subject to appropriate agreements and permissions. Once the patent is granted, we plan to explore the possibility of depositing the dataset in a public repository to enhance accessibility and reproducibility.

## Supplementary Materials

The following supporting information can be downloaded at: https://www.mdpi.com/article/10.3390/v17010014/s1, Table S1. Publicly available resource for antiviral peptide research. Table S2. Publicly available resource for assessing peptide toxicity.
